# Community Partnerships in Developing Master Plans for Aging: Lessons Learned from Santa Paula, CA

**DOI:** 10.1093/geroni/igaf122.141

**Published:** 2025-12-31

**Authors:** Ronald Berkowsky

**Affiliations:** California State University Channel Islands, Camarillo, California, United States

## Abstract

Master plans for aging (MPAs, or multisector plans for aging) provide frameworks to coordinate services (typically at the state-level) using evidence-based data to address the needs of older residents and their caregivers. Stakeholders in elder services recognize the value of multisector collaborations, as previous research has shown that these partnerships can help reduce health care utilization and cost among older residents. While several US states have implemented an MPA (e.g., California) or are currently developing an MPA, there is a growing need to identify the aging needs of local populations and to draft more county- or city-level MPAs to address these specific needs given local constraints (e.g., policies, resources). In this presentation, I describe the process of drafting an MPA for the city of Santa Paula, a predominantly Hispanic/Latinx city located in Ventura County, California. This community-engaged project marked a collaborative effort between the city, a faculty and students at a local 4-year university (i.e., California State University Channel Islands), stakeholder groups representing the particular needs of aging Santa Paula residents (e.g., the Santa Paula Senior Advisory Committee), and community-members. I describe the roles and responsibilities of those involved in this project, the methods (e.g., focus groups with local elders, quantitative surveys with city residents) and process of data collection, the results gleaned from the data, and the translation of these results to actionable insights which would form the basis of the Santa Paula MPA. Challenges and lessons learned will also be addressed throughout.

